# Aridity drives clinal patterns in leaf traits and responsiveness to precipitation in a broadly distributed Australian tree species

**DOI:** 10.1002/pei3.10102

**Published:** 2023-03-17

**Authors:** Michael J. Aspinwall, Chris J. Blackman, Chelsea Maier, Mark G. Tjoelker, Paul D. Rymer, Danielle Creek, Jeff Chieppa, Robert J. Griffin‐Nolan, David T. Tissue

**Affiliations:** ^1^ Hawkesbury Institute for the Environment Western Sydney University Penrith New South Wales Australia; ^2^ College of Forestry and Wildlife Sciences Auburn University Auburn Alabama USA; ^3^ Formation Environmental LLC Sacramento California USA; ^4^ ARC Centre of Excellence for Plant Success in Nature and Agriculture School of Natural Sciences, University of Tasmania Hobart Australia; ^5^ Faculty of Environmental Sciences and Natural Resource Management Norwegian University of Life Sciences (NMBU) Ås Norway; ^6^ Department of Biological Sciences California State University Chico California USA; ^7^ Global Centre for Land Based Innovation Western Sydney University Richmond New South Wales Australia

**Keywords:** photosynthetic capacity, phreatophyte, plasticity, stomatal behavior, thermoregulation, water‐use efficiency

## Abstract

Aridity shapes species distributions and plant growth and function worldwide. Yet, plant traits often show complex relationships with aridity, challenging our understanding of aridity as a driver of evolutionary adaptation. We grew nine genotypes of *Eucalyptus camaldulensis* subsp. *camaldulensis* sourced from an aridity gradient together in the field for ~650 days under low and high precipitation treatments. *Eucalyptus camaldulesis* is considered a phreatophyte (deep‐rooted species that utilizes groundwater), so we hypothesized that genotypes from more arid environments would show lower aboveground productivity, higher leaf gas‐exchange rates, and greater tolerance/avoidance of dry surface soils (indicated by lower responsiveness) than genotypes from less arid environments. Aridity predicted genotype responses to precipitation, with more arid genotypes showing lower responsiveness to reduced precipitation and dry surface conditions than less arid genotypes. Under low precipitation, genotype net photosynthesis and stomatal conductance increased with home‐climate aridity. Across treatments, genotype intrinsic water‐use efficiency and osmotic potential declined with increasing aridity while photosynthetic capacity (Rubisco carboxylation and RuBP regeneration) increased with aridity. The observed clinal patterns indicate that *E. camaldulensis* genotypes from extremely arid environments possess a unique strategy defined by lower responsiveness to dry surface soils, low water‐use efficiency, and high photosynthetic capacity. This strategy could be underpinned by deep rooting and could be adaptive under arid conditions where heat avoidance is critical and water demand is high.

## Graphical abstract

We grew nine genotypes of *Eucalyptus camaldulensis* sourced from an aridity gradient in the field for ~650 days under low and high precipitation treatments. We observed clinal patterns indicating that genotypes from extremely arid environments possess a unique strategy defined by greater tolerance/avoidance of dry surface soils, low water‐use efficiency, and high photosynthetic capacity. This strategy could be adaptive under arid conditions where heat avoidance is critical and water demand is high.

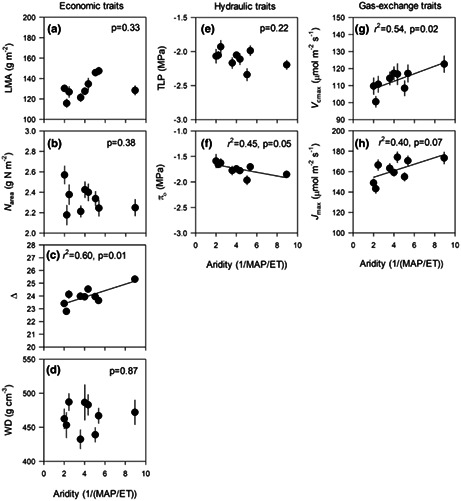

## 1 INTRODUCTION

Genecology studies have demonstrated that climate is a major driver of local adaptation and genetic differentiation within plant species (Clausen et al., 1940; Rehfeldt et al., 1999; Turesson, 1922). Manipulative experiments with genotypes from different climates have also shed light on genetic differentiation in phenotypic plasticity or physiological acclimation (Aspinwall, Fay, et al., 2017; Molina‐Montenego & Naya, 2012; Pratt & Mooney, 2013). Despite improvements in our understanding of local adaptation and phenotypic plasticity, it is unclear how climate drives genetic differentiation in plant function within different species, whether adaptation to climate results in genotypic variation in phenotypic plasticity, and whether trade‐offs emerge between a genotypes' physiological strategy and phenotypic plasticity.

Genetic differentiation in plant traits related to climate (i.e., clines) can be used to infer patterns of adaptation within species. In temperate and boreal tree species, thermal clines in growth, phenology, and physiology have been observed where populations from cold environments generally exhibit a condensed growth phase and are less productive, but invest more in leaf N, and exhibit higher photosynthetic and respiratory capacity than populations from warmer environments (Bresson et al., 2011; Dixit et al., 2022; Oleksyn et al., 1998; Soolanayakanahally et al., 2015). Much less is known about thermal clines in tree species from warmer climates where freezing is less frequent (Aspinwall, Jacob, et al., 2017; Cooper et al., 2022; Drake et al., 2017).

Clinal patterns have also been observed in genotypes sourced from rainfall or aridity gradients. However, studies often produce conflicting patterns of adaptation to aridity, complicating our understanding of aridity as a driver of adaptation. In some species, genotypes from more arid environments exhibit slower growth and conservative water use marked by tighter regulation of leaf water potential, higher intrinsic water‐use efficiency, higher leaf N, and greater resistance to drought‐induced embolism (Cregg & Zhang, 2001; Li et al., 2000; López et al., 2016; Voltas et al., 2008). Genotypes with this strategy sometimes produce smaller, thicker, and denser leaves and wood (Marchin et al., 2008; McClean et al., 2014). Leaf trait variation among species growing along aridity gradients shows similar patterns (Anderegg et al., 2020; Dong et al., 2017). In other species, genotypes from more arid environments are less conservative and exhibit lower stomatal control of leaf water potential, higher rates of leaf gas exchange, and lower water‐use efficiency (Blasini et al., 2020; Li, 1999; Zhang et al., 1993).

Conflicting patterns of adaptation to aridity could be related to the water source that plants typically access in their native environment. Species classified as obligate phreatophytes are deep‐rooted and rely entirely on groundwater to complete their life cycle (Busch et al., 1992; Canham et al., 2009). With constant access to water, these species may show high rates of leaf gas exchange, lower stomatal control of leaf water potential, and greater tolerance or avoidance of dry surface soils (Anderson et al., 1996), which may help with leaf cooling under hot conditions, albeit with greater risk of hydraulic failure if groundwater decreases (Pockman & Sperry, 2000). In contrast, facultative phreatophytes (upland species) function without access to groundwater, although there may be instances where they do access groundwater (Cooper et al., 2006; Hultine et al., 2020). These species may be more conservative in their stomatal behavior and less tolerant of dry surface soil. Phreatophytic habit (water access, stomatal strategy) can vary spatially and temporally depending upon topography and precipitation (e.g., Snyder & Williams, 2000) and may be better defined as a continuum (Hultine et al., 2020). Within species, genotypes sourced from gradients of aridity and groundwater access may also show a continuum of phreatophytic habit and stomatal behavior. Within species, selection for obligate phreatophytic habit is more likely at the most arid edge of the species distribution where groundwater, deep roots, and transpirational cooling are required (Blasini et al., 2020, 2022). Facultative habit may be more common in genotypes from less arid sites since groundwater access, deep roots, and transpirational cooling are less critical for survival. New experiments could reveal whether genetic differentiation in phreatophytic habit and stomatal behavior are shaped by adaptation to aridity.

Phenotypic differences among genotypes often depend upon growth conditions or resource availability (Bansal et al., 2015; Campbell & Sorensen, 1978; Corcuera et al., 2011), and genotypes from different environments often differ in their responsiveness to environmental change, that is, genetic variation in phenotypic plasticity (Nicotra et al., 2010). Yet, the degree to which source environment predicts genotype responsiveness to water availability remains unclear. In some shrub species, genotypes from drier locations with more interannual rainfall variability are more responsive to *increasing* soil moisture (Lázaro‐Nogal et al., 2015; Pratt & Mooney, 2013). In trees, genetic variation in responsiveness to water availability may be related to home climate or habitat (upland and lowland) in some species (Matías et al., 2014; McClean et al., 2014), but not others (Arend et al., 2011; Baquedano et al., 2008; de la Mata et al., 2014). Phreatophytic habit or stomatal behavior could partly explain why genotypes from more or less arid environments differ in responsiveness to water availability. Genotypes that invest more in roots may exhibit lower aboveground growth, but access to groundwater could facilitate higher gas‐exchange rates and greater tolerance or avoidance (i.e., lower responsiveness) of surface soil drying (Gibson et al., 1995).

To better understand genotypic variation in phreatophytic habit and responsiveness to soil moisture, it may also be important to compare and contrast plasticity of “process” and “pattern” traits. Process traits vary on short time scales and provide detailed information on energy (C, H_2_O, etc.) fluxes per unit time. Pattern traits change over longer time scales and provide a coarser view of resource investment and use (leaf economics; Volaire et al., 2020). Process and pattern traits may covary and both have been widely used to describe plant function. However, which traits and trait responses are better predictors of whole‐plant performance under changing environmental conditions is unclear, and likely depends upon growth conditions (Maréchaux et al., 2019; Moran et al., 2017).

To better understand how aridity shapes genetic differentiation and local adaptation, we sourced *Eucalyptus camaldulensis* genotypes from an aridity gradient in southeast Australia and grew them for ~650 days in the field under low and high precipitation treatments under large rainout shelters. We addressed three questions: (1) Are genotypic differences in growth, economic, hydraulic, and leaf gas‐exchange traits (including photosynthetic capacity) related to aridity at the genotype's origin? (2) Do genotypes differ in responsiveness (sensitivity) to reduced water availability, and does aridity at the genotype's origin predict genotypic variation in responsiveness to water availability? and (3) Which trait (pattern or process) responses are best at explaining differences in genotype aboveground productivity responses to precipitation? *Eucalyptus camaldulensis* is considered a phreatophyte (Merchant et al., 2007), especially in arid environments, so we hypothesized that genotypes from more arid environments would show lower aboveground productivity and higher maximum rates of leaf gas exchange, but lower responsiveness to surface drying than genotypes from less arid environments.

## 2 MATERIALS AND METHODS

### 2.1 Plant material

*Eucalyptus camaldulensis* is the most widely distributed eucalypt in Australia and is widely planted in other regions around the world. In Australia, the species is common along watercourses, especially in arid environments. The subspecies, *camaldulensis*, primarily occurs within the Murray–Darling River basin of New South Wales and Victoria, and some locations in Queensland and South Australia. Across the subspecies distribution, mean annual temperature varies from 13 to 20°C, and mean annual precipitation varies from 275 to 750 mm. Previous work has found limited genetic structure among populations within this subspecies (Dillon et al., 2015).

Nine genotypes were included in this study. A tenth genotype was planted but showed poor survival and was excluded from our analysis. Each genotype was a clone of a seedling produced by a single open‐pollinated mother tree growing naturally along an aridity gradient in southeastern Australia (Table 1). Genotypes were prepared via vegetative propagation of rooted cuttings that were established in 38 mm diameter × 210 mm tall containers filled with potting mix. Cuttings were grown in a common shade house for roughly 2 months before out‐planting in the rainout shelter experiment (see below).

**TABLE 1:** Geographic origin and long‐term average home‐climate conditions for the *Eucalyptus camaldulensis* subsp. *camaldulensis* genotypes in this study. Genotypes are listed from high to low home‐climate aridity.

Genotype	Lat (°S)	Long (°E)	*T*_max_ (°C)	*T*_mean_ (°C)	*T*_min_ (°C)	MAP (mm)	ET (mm)	Aridity 1/(MAP/ET)
20440	31.28	143.39	27.1	19.9	12.8	275	2457	8.935
19906	34.31	144.51	24.3	17.4	10.5	347	1863	5.369
15025	35.57	141.52	23.1	16.0	8.9	343	1731	5.047
19872	31.33	147.11	26.3	19.2	12.2	474	2058	4.342
20429	33.06	147.09	24.5	17.7	11.0	449	1798	4.004
15039	36.52	143.11	21.6	15.1	8.6	441	1584	3.592
19709	37.02	141.43	20.3	14.1	8.0	578	1419	2.455
19912	36.36	146.47	22.0	15.0	8.1	650	1441	2.217
19707	35.04	148.06	21.8	15.1	8.4	728	1460	2.005

*Note*: Variable descriptions: *T*_max_ = mean annual maximum air temperature, *T*_mean_ = mean annual air temperature, *T*_min_ = mean annual minimum air temperature, MAP = mean annual precipitation, ET = evapotranspiration, Aridity index = 1/(MAP/ET). Climate data are shown as averages over the period 1960 to 2014.

Average climate data (1960–2014) at the geographic origin of each genotype were generated using the *ANUClimate* 1.0 model of the Ecosystem Modelling and Scaling Infrastructure (eMAST) (Hutchinson et al., 2014). Mean annual precipitation (MAP) at the genotype's origin varied from 275 mm to 725 mm (Table 1). Mean annual temperature (MAT) at the genotype's origin varied from 14 to 20°C (Table 1). Across genotypes, MAT and MAP showed a negative correlation (*r* = −.65, *p* = .06), while MAT and mean annual evapotranspiration (ET) showed a strong positive correlation (*r* = .95, *p* < .0001). Aridity (quantified as 1/[MAP/ET]) increased with MAT (*r* = .80, *p* < .01) and decreased with MAP (*r* = −.83, *p* < .01).

### 2.2 Experimental design

This study was conducted at a rainout shelter (ROS) facility near Richmond, NSW, Australia (33.61°S, 150.74°E). Climate is warm temperate. MAT is 17°C and MAP is 800 mm. Soils are characterized by sandy loam with low organic matter content (0.7%). Six steel‐framed ROS were constructed at the site with dimensions of 12 m long × 8 m wide × 8 m tall with a roof pitch of 30° (Figure S1a). Further details on shelter design and roof function are described in Asao et al. (2020). The area under each ROS was divided into two 6 m × 8 m plots. Soil in each plot is bounded by a vertical barrier buried to a depth of 1.2 m, which limits horizontal subsoil water movement from outside the plot. A PVC sprinkler system was installed below the shelter roof and over each plot for application of separate precipitation treatments.

Eighteen randomly selected cuttings were planted in a single row (1 m spacing) around the edge of each plot to act as a buffer between treatments plots and the shelter edge. One replicate of each genotype and two randomly selected “filler” trees were planted in the “core” of each plot at 1 m × 1 m spacing (Figure S1b). Planting locations were randomized within and between plots. In total, 108 trees were included in this study (9 genotypes × 2 treatments × 6 shelters). Cuttings were planted under shelters on May 29, 2015. Average stem length at planting was 32.0 ± 8.0 cm. The cuttings received intermittent but uniform irrigation before treatments began.

Plots within each shelter were randomly assigned to a high precipitation treatment or low precipitation treatment. Treatments approximated mean annual precipitation at the wet (780 mm) and dry (275 mm) edge of the subspecies distribution. A rainfall schedule for the treatments is described in Asao et al. (2020). Briefly, irrigation in the high precipitation treatment was scheduled to replicate daily rainfall of a year (1971) with MAP similar to average MAP at the wettest site during 1960–2014. Irrigation in the low precipitation treatment followed the same schedule, but with reduced amounts to approximate MAP of the driest site. Treatments began on November 15, 2015. We permanently ceased rainfall in the low precipitation treatment 3 months after starting the treatments (February 20, 2016) because soil moisture was only slightly different between treatments. The experiment ended when the trees were harvested; roughly 1 year after ceasing rainfall in the low precipitation treatment. The original rainfall schedule, actual daily amounts (in mm) applied to each plot, and detailed notes on treatment application are provided in Dataset S1.

Soil volumetric water content (VWC, m^3^ m^−3^) in each plot was continuously measured using four soil moisture probes (Campbell Scientific): one at 30 cm depth, two at 55 cm depth, and one at 80 cm depth. Air temperature (*T*_air_) and relative humidity (RH) in the center of each shelter were measured using a temperature–relative humidity probe (HMP45C, Campbell Scientific Inc.). Daily minimum, mean, and maximum air temperature and vapor pressure deficit are shown in Figure S3.

### 2.3 Growth, biomass, and economic traits

Stem basal diameter at 5 cm stem length (*D*, cm) and stem length (*L*, cm) were measured on each tree each month (*n* = 21 time points). Stem volume (*V*, cm^3^) was estimated based on the volume of a cone: π × (*D*/2)^2^ × (*L*/3). Trees were harvested the week of 6 March 2017, roughly 650 days (1.75 years) after the treatments began. Average *L* at harvest was >5 m. At harvest, diameter at breast height (1.3 m, DBH) was recorded and the shoot of each tree was cut at ground level. The shoot was separated into leaves, branches, and stem. Leaf, branch, and stem dry mass (DM), average leaf size (LS, cm^2^), total tree leaf area (LA, m^2^), wood density (WD, g m^−3^), stem Huber value (HV), leaf nitrogen per unit area (*N*_area_, g N m^−2^), and discrimination of leaf ^13^C relative to the atmosphere (Δ) were determined as described in Methods S1. Δ reflects the ratio of intercellular CO_2_ (*C*_i_) to atmospheric CO_2_ (*C*_a_) and is negatively associated with intrinsic water‐use efficiency (ratio of photosynthesis to stomatal conductance).

### 2.4 Leaf water potential and gas exchange

Predawn and midday leaf water potential (*Ψ*_pd_ and *Ψ*_md_) were measured on 1–3 mature, fully expanded upper canopy leaves per tree at nine timepoints (dates) using a pressure chamber (PMS Instruments). *Ψ*_pd_ and *Ψ*_md_ were measured at the same timepoints as leaf gas‐exchange measurements (described below), and three additional timepoints (June 9, 2016, February 9, 2017, and February 22, 2017). Leaves for *Ψ*_pd_ measurements were collected roughly 30 min before sunrise and leaves for *Ψ*_md_ were collected at 12:30 h local time (±30 min). Leaves were placed in sealed ziplock bags containing moist paper and were kept in the dark for at least 30 min before measuring.

Leaf‐level net photosynthesis CO_2_ response (*A‐Ci*) measurements were made at five timepoints (February 22, 2016, May 4, 2016, September 15, 2016, January 10, 2017, March 9, 2017) using four cross‐calibrated portable infrared gas analyzers (LI‐6400XT, Li‐Cor Inc.) fitted with a 2 × 3 cm cuvette head and a red and blue LED light source. *A‐Ci* measurements were made on mature, fully expanded, upper canopy leaves. For all measurements, light conditions within the cuvette were controlled at a photosynthetic photon flux density of 1800 μmol m^−2^ s^−1^. Flow rate was held constant at 500 μmol s^−1^. Block temperature was set at 25°C for all measurements although leaf temperature varied among sampling dates according to ambient air temperature and water fluxes from the leaves. Relative humidity in the chamber was controlled near ambient external conditions, but also varied depending upon water vapor fluxes from the leaf. Each *A‐Ci* curve began with steady‐state measurements of light‐saturated net photosynthesis (*A*_net_), stomatal conductance to water vapor (*g*_s_), and intercellular CO_2_ concentration (*C*_i_) at a chamber reference CO_2_ of 420 μmol mol^−1^. Following steady‐state measurements, *A‐Ci* curves were produced by measuring *A*_net_ at a series of reference CO_2_: 230, 150, 100, 50, 420, 650, 800, 1200, and 1500 μmol mol^−1^. Each *A‐Ci* curve was parameterized using the Farquhar model of C_3_ photosynthesis (Farquhar et al., 1980). The model estimates the maximum rate of Rubisco carboxylation (*V*_cmax_) and the rate of electron transport for RuBP regeneration (*J*_max_). We did not measure mesophyll conductance such that estimates of *V*_cmax_ and *J*_max_ are “apparent” rates that reflect both biochemical limitations of photosynthesis and mesophyll conductance. The model was fit using nonlinear least squared parameter estimation in SAS v9.3 (PROC NLIN; SAS Institute Inc. 2010). We also measured *A*_net_ and *g*_s_ at one additional timepoint (July 14, 2016, six timepoints total), with the same approach for controlling light, flow, temperature, and humidity.

Measurements of leaf dark respiration were carried out on the same leaves 1–3 days after photosynthetic measurements. At each time point (*n* = 6), sampling occurred randomly among genotypes and treatments. Leaves were collected ~2 h after sunset, placed in sealed plastic bags with moist paper, and transported to a room set to approximately 25°C. Leaf area of sampled leaves was determined with a leaf area meter (LI‐3100C; Li‐Cor Inc.). Leaf respiration per unit area at 25°C (*R*_area_^25^, μmol m^−2^ s^−1^) was determined by placing entire leaves in large gas‐exchange chamber (LI‐6400‐22L; Li‐Cor) attached to infrared gas analyzers. Block temperature was maintained at 25°C, reference [CO_2_] was fixed at 400 μmol mol^−1^, and flow rate was set at 500 μmol s^−1^. Leaves were kept in darkness by covering the chambers with a dark cloth. All *R*_area_^25^ measurements were made within ~4 h of leaf collection. Following measurements, leaves were placed in envelopes and dried at 70°C for 72 h. Leaf dry mass per unit area (LMA) of the gas‐exchange leaves was calculated as the ratio of leaf dry mass (g) to leaf area multiplied by 0.0001. Rates of respiration per unit dry mass (*R*_mass_^25^, nmol g^−1^ s^−1^) were determined by multiplying *R*_area_^25^ by 1000 and dividing the product by LMA.

### 2.5 Leaf hydraulic traits

Leaf hydraulic vulnerability was assessed in February 2017 using a single‐point approach (Lucani et al., 2018), where leaf hydraulic conductance (*K*_leaf_, mmol m^−2^ MPa^−1^ s^−1^) was measured at a reference water potential associated with hydraulic decline. This allowed us to compare *K*_leaf_ across genotypes and treatments without determining the response of *K*_leaf_ to the full range of water potentials. We targeted a reference water potential of −4 MPa to make comparisons of dehydrated *K*_leaf_ and relative hydraulic vulnerability. This level of water stress was associated with incipient *K*_leaf_ decline in a series of unpublished leaf hydraulic vulnerability curves for *E. camaldulensis*. The method for determining dehydrated *K*_leaf_ is fully described in Methods S2.

Pressure–volume analysis was performed in February 2017 using standard procedures (Tyree & Hammel, 1972). We selected one fully expanded leaf from the upper canopy of the same trees used for *K*_leaf_ measurements. For each leaf, the turgor loss point (TLP), osmotic potential (π_o_), bulk modulus of elasticity (ε), and the slope of the pre‐ and post‐turgor loss relationship between relative water content and Ψ were determined. A complete list of all variables and traits considered in this study is included in Table 2.

**TABLE 2:** List of all variables (or traits) included in this study, including their abbreviations, units, and classification as a “pattern” or “process” trait.

Variable/trait	Abbreviation/symbol	Units	Pattern or process trait
Stem diameter	Stem *D*	cm	Pattern
Diameter at breast height	DBH	cm	Pattern
Stem length	Stem *L*	cm	Pattern
Leaf dry mass	Leaf DM	g	Pattern
Total leaf area	Total LA	m^2^	Pattern
Huber value	HV	Unitless (×10^4^)	Pattern
Stem dry mass	Stem DM	g	Pattern
Branch dry mass	Branch DM	g	Pattern
Aboveground dry mass	Aboveground DM	g	Pattern
Average leaf size	LS	cm^2^	Pattern
Leaf mass per unit area	LMA	g m^−2^	Pattern
Leaf N per unit area	*N*_area_	g N m^−2^	Pattern
Discrimination of leaf ^13^C relative to the atmosphere	Δ	Unitless	Pattern
Wood density	WD	g cm^−3^	Pattern
Predawn water potential	*Ψ*_pd_	MPa	Process
Midday water potential	*Ψ*_md_	MPa	Process
Dehydrated leaf hydraulic conductance	Dehyd. *K*_leaf_	mmol m^−2^ s^−1^ MPa^−1^	Process
Turgor loss point	TLP	MPa	Pattern/Process
Osmotic potential	π_o_	MPa	Pattern/Process
Net photosynthesis	*A*_net_	μmol m^−2^ s^−1^	Process
Stomatal conductance to water vapor	*g*_s_	mol m^−2^ s^−1^	Process
Maximum rate of Rubisco carboxylation	*V*_cmax_	μmol m^−2^ s^−1^	Process
Rate of electron transport for RuBP regeneration	*J*_max_	μmol m^−2^ s^−1^	Process
Leaf dark respiration per unit area at 25 °C	*R*_area_^25^	μmol m^−2^ s^−1^	Process
Leaf dark respiration per unit mass at 25 °C	*R*_mass_^25^	μmol m^−2^ s^−1^	Process

### 2.6 Data analysis

All analyses were performed in SAS v9.3 (SAS Institute Inc. 2010). A two‐sample *t*‐test was used to compare soil moisture (VWC) between treatments after the treatments were initiated. We used mixed‐model analysis of variance (PROC MIXED) to test the main and interactive effects of genotype (G) and precipitation treatment (P) on each response variable (i.e., trait). Measurement “date” was considered a random effect for variables that were repeatedly measured over time (e.g., *Ψ*_pd_, *A*_net_, and *R*_area_^25^) since sampling occurred somewhat randomly and the effects of “date” were not the focus of this study. Rainout shelters were treated as blocks and were also considered a random effect. If the interaction between G and P (G × P) was significant, this indicated that (1) genotypic differences were dependent upon precipitation and (2) responsiveness (plasticity) to precipitation differed among genotypes. When G or G × P effects were significant, we used Tukey's adjustment for *post hoc* comparison of genotype means. When G × P was significant for a particular trait, we examined relationships (PROC REG) between genotype means and aridity separately for each treatment, as well as relationships between genotype responsiveness to reduced (low) precipitation and aridity. For each variable, genotype responsiveness was calculated as the relative change in trait X, that is, responsiveness = 100 × ([X_L_ – X_H_]/X_H_), where X_L_ is the genotype mean value under low precipitation and X_H_ is the genotype mean value under high precipitation.

## 3 RESULTS

### 3.1 Soil moisture

Averaged across all timepoints and shelters, daily mean VWC at all depths was significantly higher under high precipitation than low precipitation (Figure S2). At 30 cm depth, daily mean VWC averaged 0.089 ± 0.03 m^3^ m^−3^ (standard deviation) under high precipitation and 0.061 ± 0.03 m^3^ m^−3^ under low precipitation. When daily mean VWC at 30 cm was summed across all dates and shelters, it was ~50% higher under high precipitation than low precipitation. At 50 cm depth, daily mean VWC averaged 0.082 ± 0.04 m^3^ m^−3^ under high precipitation and 0.061 ± 0.04 m^3^ m^−3^ under low precipitation. At 80 cm depth, average VWC averaged 0.089 ± 0.04 m^3^ m^−3^ under high precipitation and 0.073 ± 0.04 m^3^ m^−3^ under low precipitation (Figure S2). Daily mean VWC in the low precipitation treatment increased slightly during the middle of study, likely due to subsurface water movement from rainfall outside the plots.

### 3.2 Whole‐tree growth and productivity

**FIGURE 1:**
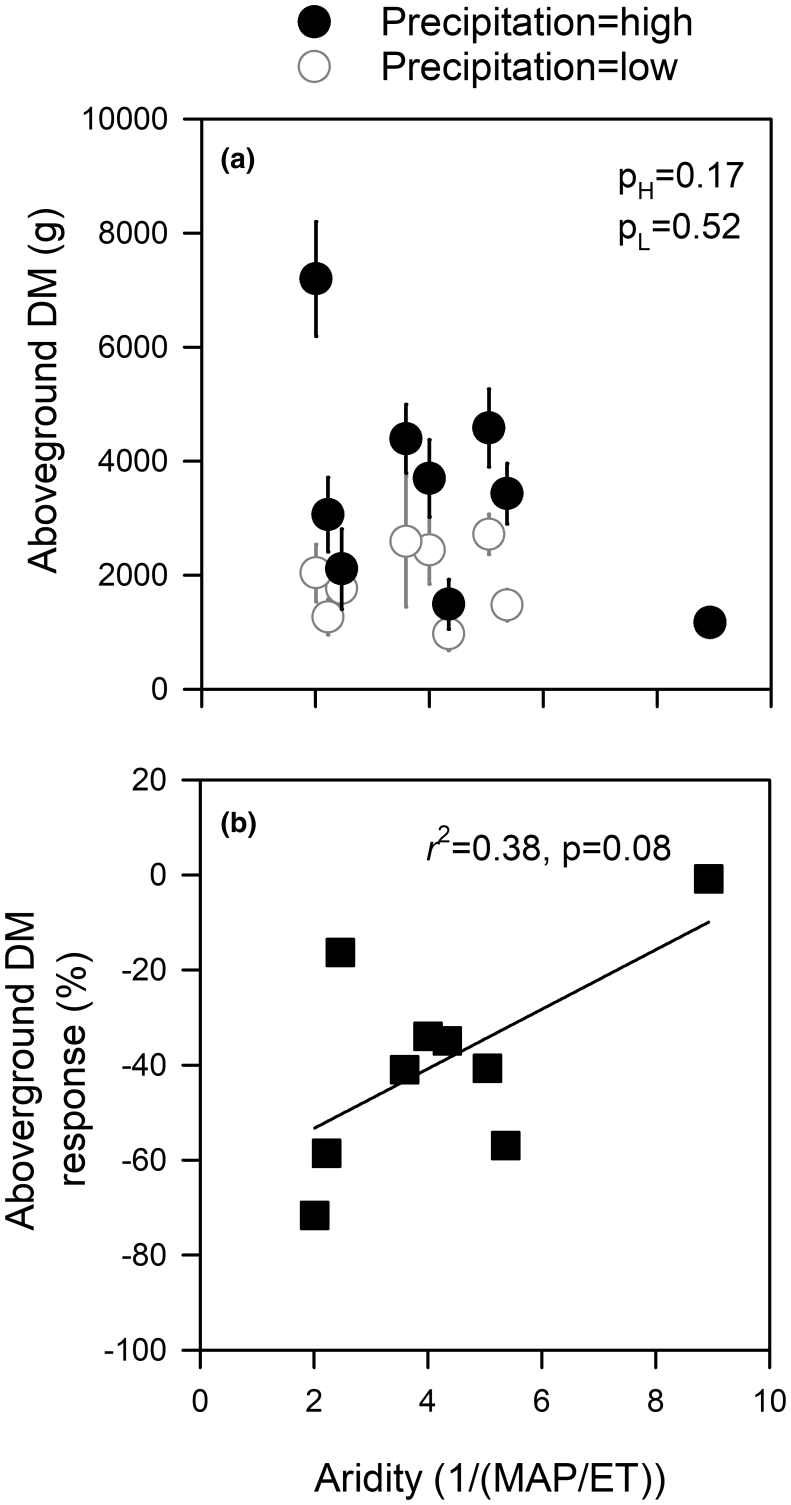
(a) Mean (±standard error, *n* = 6) values of aboveground dry mass (DM) for nine *Eucalyptus camaldulensis* genotypes grown under high and low precipitation treatments in a common garden in relation to the genotype's home‐climate aridity (low aridity values = less arid, high aridity values = more arid). In (a), p_H_ is the *p*‐value for the relationship between aridity and aboveground DM under high precipitation, while p_L_ is the *p*‐value for the relationship between aridity and aboveground DM under high precipitation. (b) Genotype aboveground DM response to reduced precipitation calculated as Response (%) = 100 × ([X_L_ – X_H_]/X_H_), where X_L_ is the genotype mean under low precipitation and X_H_ is the genotype mean under high precipitation.

Most growth and biomass variables showed a significant G × P interaction (Table 3). For simplicity, we focus on total aboveground mass since stem *D*, DBH, and biomass components all showed G × P interactions of similar magnitude (Table 3, Figure S4). Under high precipitation, genotype differences in aboveground biomass were large. For instance, the genotype from the least arid environment showed >500% higher aboveground mass than the genotype from the most arid environment (*p* < .0001, Figure 1a). Under low precipitation, genotype differences in aboveground biomass were not significant (Figure 1a). Mean aboveground mass of individual genotypes was not related to aridity under either precipitation treatment (Figure 1a; Figure S4). Patterns of tree growth (stem *V*) over time are shown in Figure S5.

**TABLE 3:** Analysis of variance for genotype (G), precipitation treatment (P), G × P effects on *Eucalyptus camaldulensis* subsp. *camaldulensis* growth, and leaf traits.

	Genotype (G)		Precipitation (P)		G × P		Precipitation means (±standard error)	
	*df*	*F*	*df*	*F*	*df*	*F*	Low P	High P
*Growth and biomass traits*								
Stem *D* (cm)	8,80	7.8***	1,5	48.6**	8,80	3.2**	4.86 ± 0.2	6.40 ± 0.3
DBH (cm)	8,80	7.7***	1,5	30.4**	8,80	3.3**	2.82 ± 0.1	3.87 ± 0.2
Stem *L* (cm)	8,80	8.7***	1,5	29.5**	8,80	1.6	483 ± 14	607 ± 18
Leaf DM (g)	8,80	8.3***	1,5	19.6**	8,80	3.3**	430 ± 47	691 ± 67
Total LA (m^2^)	8,80	9.0***	1,5	27.7**	8,80	3.8**	3.17 ± 0.4	5.55 ± 0.5
HV (×10^4^)	8,80	1.4	1,5	0.5	8,80	0.7	3.61 ± 0.1	3.46 ± 0.1
Stem DM (g)	8,80	10.3***	1,5	58.2**	8,80	4.4**	1093 ± 99	2159 ± 183
Branch DM (g)	8,80	9.2***	1,5	33.1**	8,80	4.0**	305 ± 41	612 ± 66
Aboveground DM (g)	8,80	10.0***	1,5	44.8**	8,80	4.3**	1827 ± 183	3461 ± 310
*Economic traits*								
LS (cm^2^)	8,80	62.9***	1,5	5.0†	8,80	1.6	20.5 ± 0.9	23.1 ± 0.8
LMA (g m^−2^)	8,80	11.2***	1,5	21.9**	8,80	1.0	138 ± 2	123 ± 2
*N*_area_ (g N m^−2^)	8,79	3.1**	1,5	0.1	8,79	0.7	2.32 ± 0.04	2.34 ± 0.04
Δ	8,79	16.8***	1,5	28.2**	8,79	0.3	23.56 ± 0.1	24.36 ± 0.1
WD (g cm^−3^)	8,80	2.2*	1,5	33.7**	8,80	1.0	491 ± 8	439 ± 6
*Hydraulic traits*								
*Ψ*_pd_ (MPa)^ln^	8,40	1.6	1,5	16.0*	8781	4.4***	−0.57 ± 0.02	−0.37 ± 0.01
*Ψ*_md_ (MPa)^ln^	8,40	8.2***	1,5	10.8*	8782	2.8**	−1.76 ± 0.02	−1.49 ± 0.02
Dehyd. *K*_leaf_ (mmol m^−2^ s^−1^ MPa^−1^)	7,27	3.2*	1,2	1.7	7,27	2.1†	13.0 ± 0.5	11.4 ± 0.6
TLP (MPa)	8,32	4.2**	1,2	12.9†	8,32	1.2	−2.20 ± 0.04	−2.00 ± 0.03
π_o_ (MPa)	8,32	3.0*	1,2	7.4	8,32	1.6	−1.80 ± 0.04	−1.68 ± 0.03
*Gas‐exchange traits*								
*A*_net_ (μmol m^−2^ s^−1^)	8,40	2.2*	1,5	15.8*	8499	2.3*	19.4 ± 0.4	22.1 ± 0.3
*g*_s_ (mol m^−2^ s^−1^)^ln^	8,40	2.2*	1,5	32.3**	8499	2.9**	0.386 ± 0.02	0.612 ± 0.02
*V*_cmax_ (μmol m^−2^ s^−1^)^ln^	8,32	2.6*	1,4	0.1	8403	0.5	115 ± 2.6	111 ± 1.8
*J*_max_ (μmol m^−2^ s^−1^)^ln^	8,32	4.8**	1,4	2.7	8403	0.7	155 ± 1.9	169 ± 2.5
*R*_area_^25^ (μmol m^−2^ s^−1^)^ln^	8,40	1.9†	1,5	2.6	8502	1.7†	1.05 ± 0.02	1.15 ± 0.03
*R*_mass_^25^ (nmol g^−1^ s^−1^)^ln^	8,40	1.8	1,5	6.1†	8499	1.3	8.39 ± 0.2	10.23 ± 0.3

*Note*: Variable descriptions: Stem D, basal diameter at 5 cm of stem length; DBH, diameter at breast height; Stem L, stem length; Leaf DM, leaf dry mass; Total LA, total leaf area; HV, Huber value; Stem DM, stem dry mass; Branch DM, branch dry mass; Aboveground DM, aboveground dry mass; Average LS, average leaf size; LMA, leaf dry mass per unit area; *N*_area_, leaf N per unit area; LMA, leaf dry mass per unit leaf area; *N*_area_, leaf N per unit area; Δ, discrimination of ^13^C relative the atmosphere; WD, wood density; *Ψ*_pd_, predawn water potential; *Ψ*_md_, midday water potential; Dehyd. *K*_leaf_, leaf hydraulic conductance at dehydrated state; TLP, turgor loss point; π_o_, osmotic potential at full turgor; *A*_net,_ net photosynthesis; *g*_s_, stomatal conductance to water vapor; *V*_cmax_, Rubisco carboxylation; *J*_max_, electron transport for RuBP regeneration; *R*_area_^25^, leaf dark respiration per unit area at 25°C; *R*_mass_^25^, leaf dark respiration per unit mass at 25°C. “ln” indicates that variable was log‐transformed to fulfill assumptions of normality.

Numerator and denominator degree of freedom (*df*) and *F‐*values are presented for each variable and effect. *F*‐values denoted with “***”, “**”, “*”, and “†” are significant at *p* < .001, *p* < .01, *p* < .05, and *p* < .10 respectively.

Responsiveness of aboveground mass to reduced precipitation differed among genotypes. The genotype from the least arid environment showed the largest reduction in aboveground mass (−72%) in response to reduced precipitation while the genotype from the most arid environment showed the smallest negative response to reduced precipitation (−0.9%). Across all genotypes, we found a weak relationship between aridity and aboveground mass response to reduced precipitation where genotypes from less arid environments showed larger negative responses to reduced precipitation than genotypes from more arid environments (Figure 1b).

Stem length and HV showed no G × P interaction. Stem length differed between treatments and among genotypes (Table 3). On average, stem length was 20% lower in the low precipitation treatment than the high precipitation treatment (Table 3), and genotypes differed in stem length by up to 52% (Figure S4). Genotypic differences in stem length were not associated with aridity (*p* = .24). HV was similar across genotypes and treatments and averaged 3.54 ± 0.09 × 10^4^.

### 3.3 Leaf hydraulic traits

**FIGURE 2:**
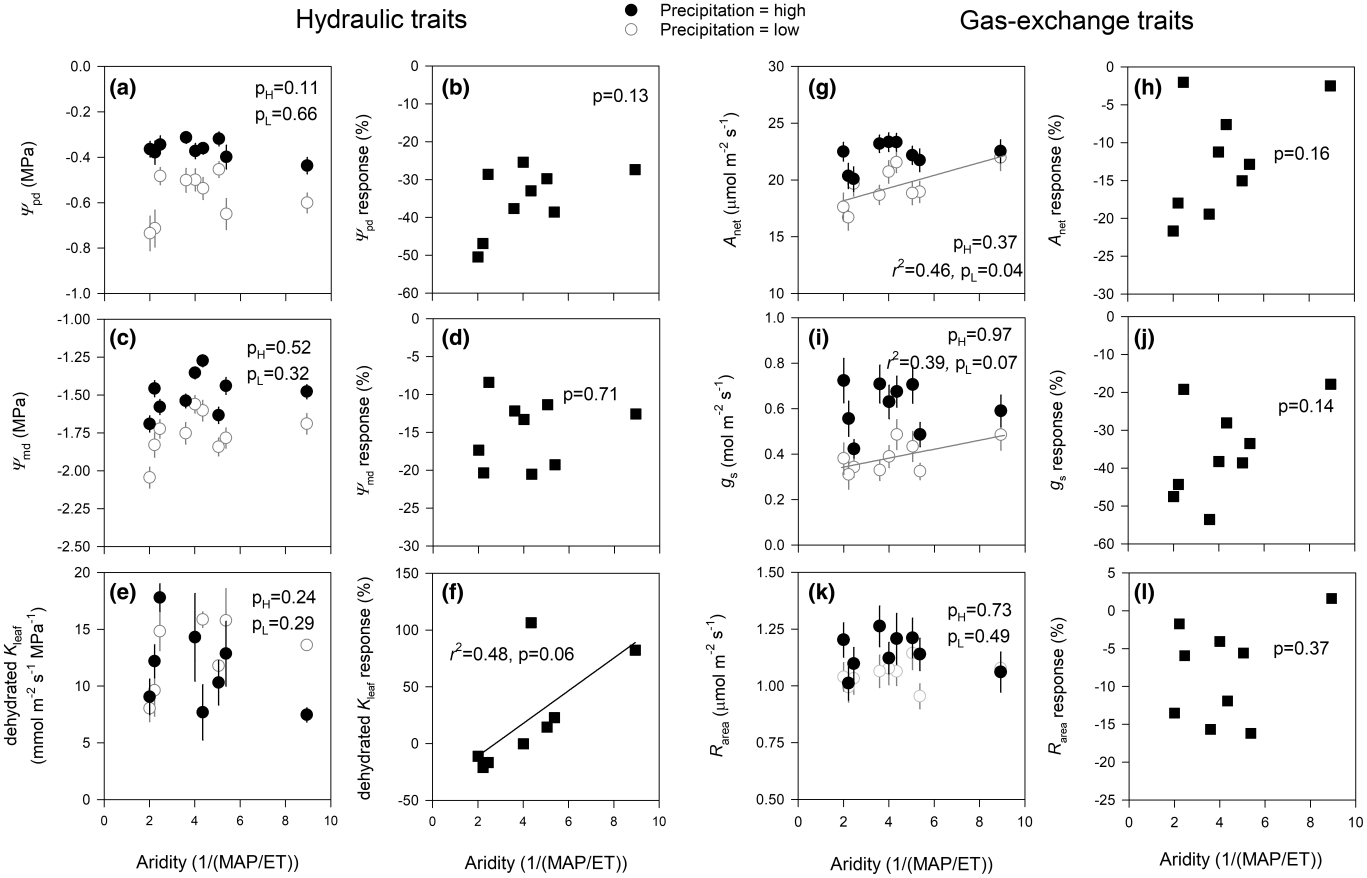
Mean (±standard error, *n* = 6) values for leaf hydraulic (a, c, and e) and gas‐exchange traits (g, i, and k) for nine *Eucalyptus camaldulensis* genotypes grown under high and low precipitation treatments in a common garden. These traits showed evidence of genotype × precipitation interactions. Means are plotted against the genotype's home‐climate aridity (low aridity values = less arid, high aridity values = more arid). Regression lines and coefficients of determination (*r* ^2^) are show when trait–aridity relationships were significant at *p* < .10. Genotype trait responses to reduced precipitation in relation to home‐climate aridity are also shown (panels b, d, f, h, j, and l). Genotype responses were calculated as: Response (%) = 100 × ([X_L_ – X_H_]/X_H_), where X_L_ is the genotype mean under low precipitation and X_H_ is the genotype mean under high precipitation.

Significant G × P interactions were observed for *Ψ*_pd_, *Ψ*_md_, and dehydrated *K*_leaf_ (Table 3). Averaged across all timepoints, genotypes showed no differences in *Ψ*_pd_ under high precipitation (average *Ψ*_pd_ = −0.57 MPa, Table 3). Under low precipitation, genotype mean *Ψ*_pd_ varied from −0.73 ± 0.08 to −0.45 ± 0.03 MPa. While these values, which are averages across all timepoints, are relatively high, we note that *Ψ*_pd_ values under low precipitation were considerably lower at some timepoints (lowest values ranging from −2.8 MPa to –1.4 MPa), including January 10 2017, February 9, 2017, March 9, 2017. Genotype *Ψ*_pd_ was not related to aridity under either precipitation treatment. Responsiveness of *Ψ*_pd_ to reduced precipitation differed among genotypes, with the largest reductions in *Ψ*_pd_ observed in genotypes 19,707 (−50%, *p* < .0001), 19,912 (−49%, *p* < .0001), and 19,906 (−39%, *p* = .006, Figure 2a). The response of *Ψ*_pd_ to precipitation for individual genotypes was not associated with aridity, although genotypes from more arid environments tended to show smaller reductions in *Ψ*_pd_ under low precipitation (*p* = .13, Figure 2b).

Genotypes varied in *Ψ*_md_ in both treatments. Under high precipitation, genotype mean *Ψ*_md_ varied from −1.69 ± 0.05 to −1.27 ± 0.04. Under low precipitation, genotype mean *Ψ*_md_ varied from −2.05 ± 0.07 to −1.56 ± 0.06 MPa (Figure 2c). Genotypic variation in *Ψ*_md_ was not related to aridity under either precipitation treatment. Responsiveness of *Ψ*_md_ to reduced precipitation differed among genotypes with the largest reductions in *Ψ*_md_ observed in genotypes 19,707 (−17%, *p* = .07), 19,872 (−21%, *p* = .02), 19,912 (−20%, *p* = .02), and 19,906 (−19%, *p* = .01, Figure 2d). Responsiveness of *Ψ*_md_ to reduced precipitation for individual genotypes was not associated with aridity (Figure 2d).

Although a significant G × P interaction was detected for dehydrated *K*_leaf_, *post hoc* comparisons showed no differences in dehydrated *K*_leaf_ among genotypes in either precipitation treatment, and genotype means for dehydrated *K*_leaf_ were not related to aridity in either treatment (Figure 2e). Although responsiveness of dehydrated *K*_leaf_ to reduced precipitation varied among genotypes, this variation was not significant for most genotypes. Nonetheless, variation in the mean response was associated with aridity where genotypes from less arid environments showed little change in dehydrated *K*_leaf_, while genotypes from more arid environments showed larger *increases* in dehydrated *K*_leaf_ with reduced precipitation (Figure 2). One genotype (19872) was an exception to the general pattern (Figure 2f, studentized residual = 2.4), and its removal strengthened the relationship between aridity and dehydrated *K*_leaf_ response to reduced precipitation (*r* ^2^ = 0.97, *p* < .0001).

**FIGURE 3:**
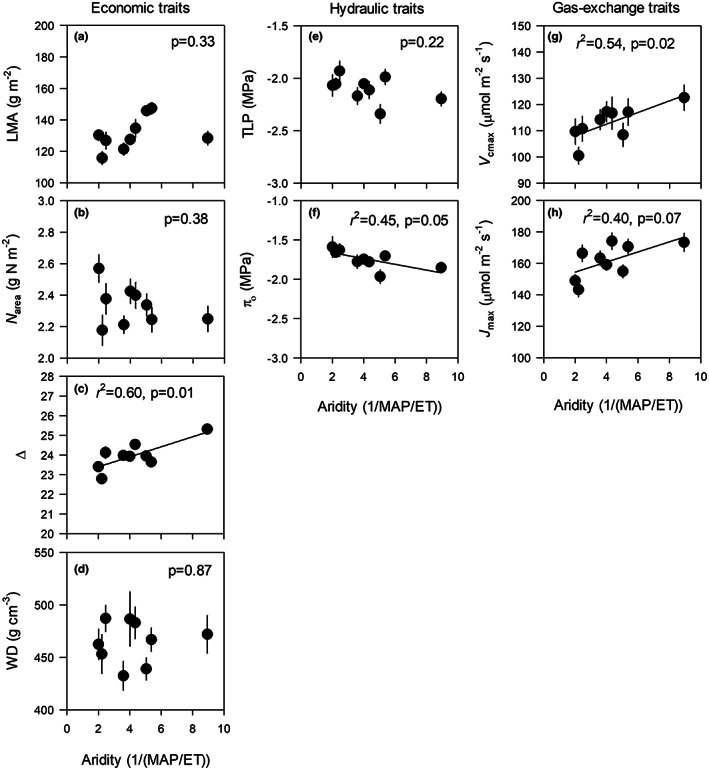
Relationships between genotype means (±standard error, *n* = 12) for several economic (a, b, c, and d), hydraulic (e and f), and gas‐exchange (g and h) traits and genotype home‐climate aridity (low aridity values = less arid, high aridity values = more arid). These traits showed no evidence of genotype × precipitation interactions (unlike traits shown in Figures 1 and 2). Plotted means are average values across treatments and timepoints (in the case of *V*_cmax_ and *J*_max_). Regression lines and coefficients of determination (*r* ^2^) are shown when trait–aridity relationships are significant at *p* < .10.

TLP and π_o_ differed among genotypes. Across genotypes, mean TLP varied from −2.33 ± 0.09 to −1.93 ± 0.09 MPa (Figure 3e). This variation was not associated with aridity (Figure 3e). Mean π_o_ varied from −1.96 ± 0.09 to −1.59 ± 0.13 MPa across genotypes and declined with increasing aridity (Figure 3f). TLP was lower (0.2 MPa, −10%) under low precipitation than high precipitation, but π_o_ was similar between treatments and averaged −1.74 ± 0.03 MPa (Table 3).

### 3.4 Economic traits

Most economic traits differed between precipitation treatments and among genotypes, but none showed significant G × P interactions (Table 3). Average LS was slightly lower under low precipitation, and LMA and WD were higher under low precipitation than high precipitation (+12% and +12% respectively, Table 3). Discrimination of ^13^C (Δ) was lower under low precipitation (−3%), indicating higher intrinsic water‐use efficiency. *N*_area_ was similar between precipitation treatments (Table 3).

Average LS varied ~twofold across genotypes (14.8 ± 0.8 to 31.8 ± 1.5 cm^2^), but this variation was not associated with aridity (*p* = .13, Figure S6). LMA varied from 116 ± 4.1 to 147 ± 3.6 g m^−2^ across genotypes. WD varied from 432 ± 13.8 to 487 ± 12.5 g cm^−3^ across genotypes. Genotypic differences in LMA and WD were not associated with aridity (Figure 3a,d). Across genotypes, Δ varied from 22.8 ± 0.2 to 25.3 ± 0.2 and increased with aridity, indicating that intrinsic water‐use efficiency was lower in genotypes from more arid environments (Figure 3c). *N*_area_ varied from 2.18 ± 0.1 to 2.57 ± 0.1 g N m^−2^ across genotypes and was not related to aridity (Figure 3d).

### 3.5 Leaf gas‐exchange traits

A significant G × P interaction was observed for *A*_net_ and *g*_s_. *R*_area_^25^ also showed a weak G × P interaction. *A*_net_ did not differ among genotypes under high precipitation (average *A*_net_ = 22.1 μmol m^−2^ s^−1^, Table 3), but ranged from 16.7 ± 1.1 to 22.0 ± 1.1 μmol m^−2^ s^−1^ across genotypes under low precipitation; a relative difference of 32%. Under low precipitation, genotype mean *A*_net_ increased with aridity (Figure 2g). Responsiveness of *A*_net_ to reduced precipitation differed among genotypes with significant reductions in *A*_net_ in genotypes 15,039 (−19%, *p* = .02), 19,707 (−22%, *p* = .006), and 19,912 (−18%, *p* = .02, Figure 2h). Across genotypes, the response of *A*_net_ to reduced precipitation was not correlated with aridity (*p* = .16), although larger negative responses were generally observed in genotypes from less arid environments (Figure 2h).

We observed similar patterns in the *g*_s_ results; *g*_s_ did not differ among genotypes under high precipitation (mean *g*_s_ = 0.612 mol m^−2^ s^−1^, Table 3). However, under low precipitation g_s_ varied from 0.310 ± 0.06 to 0.486 ± 0.07 mol m^−2^ s^−1^ across genotypes (Figure 2i). Under low precipitation, genotype mean *g*_s_ increased with aridity (Figure 2i). Responsiveness of *g*_s_ to reduced precipitation differed among genotypes with significant reductions observed in genotypes 15,025 (−39%, *p* = .02), 15,039 (−54%, *p* < .0001), 19,707 (−48%, *p* < .0001), 19,912 (−44%, *p* < .001), and 20,429 (−38%, *p* = .05, Figure 2j). Like *A*_net_, genotype *g*_s_ responses to reduced precipitation were not correlated with aridity (*p* = .14), although larger negative responses were generally observed in genotypes from less arid environments (Figure 2j).

Although *R*_area_^25^ showed a weak G × P interaction, *post hoc* comparisons revealed that *R*_area_^25^ did not differ among genotypes in either treatment. Furthermore, the response of *R*_area_^25^ to reduced precipitation did not differ among genotypes (Figure 2k). Averaged across treatments and genotypes, mean *R*_area_^25^ was 1.10 ± 0.02 μmol m^−2^ s^−1^. *R*_mass_^25^ was similar among genotypes and showed no G × P interaction. *R*_mass_^25^ was lower (−18%) under low precipitation than high precipitation.

*V*_cmax_ and *J*_max_ showed no G × P interaction and did not differ between treatments. However, both variables differed among genotypes. Across genotypes, *V*_cmax_ varied from 101 ± 3.2 to 123 ± 4.8 μmol m^−2^ s^−1^. *J*_max_ varied from 143 ± 4.5 to 174 ± 5.2 μmol m^−2^ s^−1^ across genotypes (Figure 3g,h). Genotype means for *V*_cmax_ and *J*_max_ increased with aridity, indicating a positive genetic correlation between photosynthetic capacity and aridity (Figure 3g,h).

### 3.6 Productivity response–trait response relationships

**FIGURE 4:**
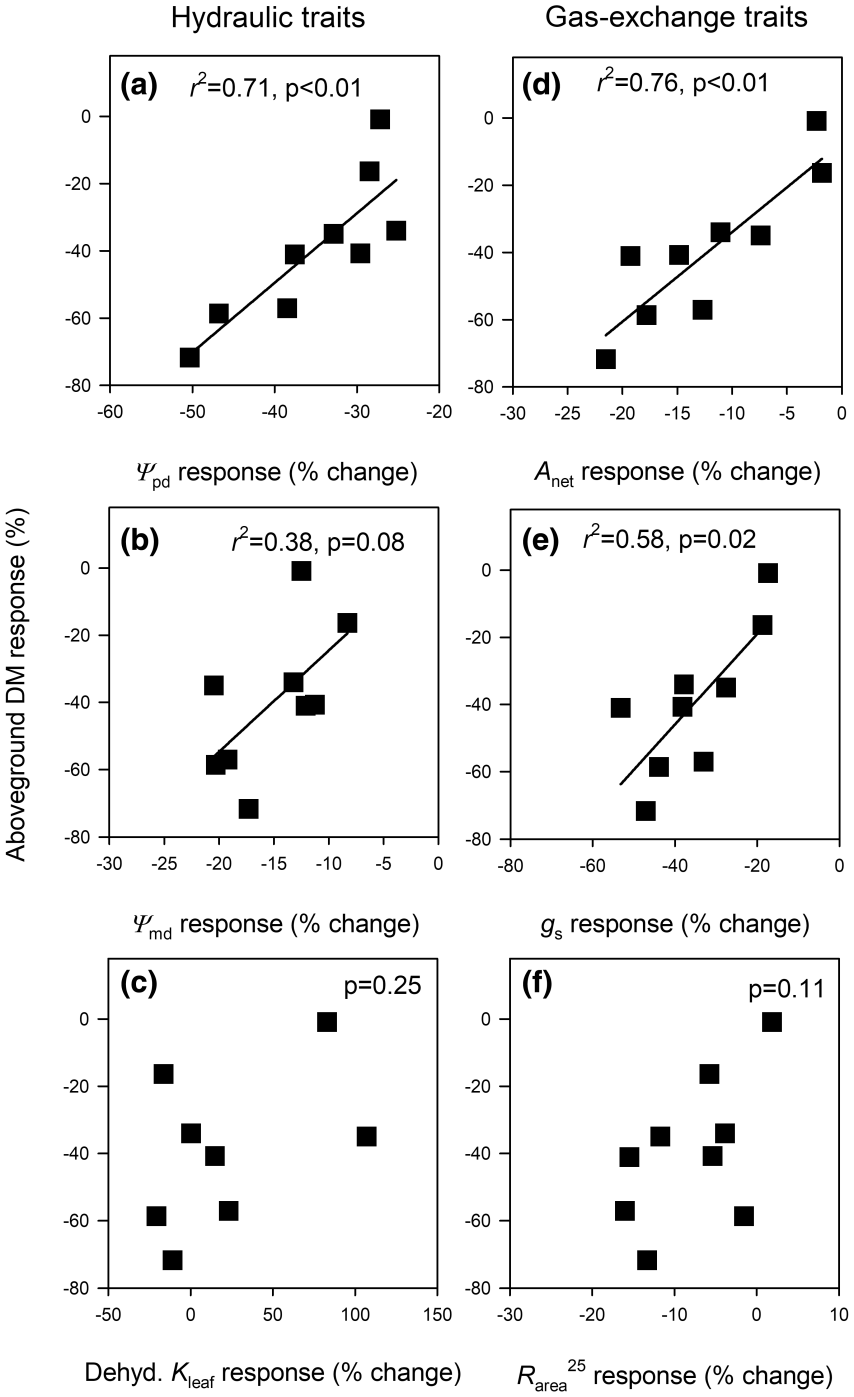
Relationships between genotype leaf trait (hydraulic, gas exchange) responses (% change) to reduced precipitation and genotype aboveground DM responses to reduced precipitation. Regression lines and coefficients of determination (*r* ^2^) are shown when relationships are significant at *p* < .10.

G × P interactions were more common for leaf hydraulic and gas‐exchange traits (mostly process traits) than economic traits (pattern traits). Thus, we focused on examining relationships between genotype hydraulic and gas‐exchange responses to reduced precipitation, and genotype productivity (aboveground dry mass) responses to reduced precipitation. Across genotypes, *Ψ*_pd_, *Ψ*_md_, *A*_net_, and *g*_s_ responses to reduced precipitation were strongly correlated (with exception to midday) with productivity responses to reduced precipitation (Figure 4). Genotypes that showed smaller reductions in these traits also showed small reductions in aboveground dry mass responses under low precipitation. Genotype‐dehydrated *K*_leaf_ and *R*_area_^25^ responses to reduced precipitation were not correlated with genotype productivity responses (Figure 4).

## 4 DISCUSSION

We tested whether *Eucalyptus camaldulensis* genotypes sourced from an aridity gradient show clinal patterns in multiple traits and responsiveness to experimental precipitation treatments. We found that aridity predicted genotype responses to precipitation, with more arid genotypes showing lower responsiveness to reduced precipitation than less arid genotypes. Across genotypes, lower responsiveness of leaf water potential and gas exchange to low precipitation was associated with smaller reductions in aboveground biomass. Therefore, process trait responses were better predictors of genotype growth responses to precipitation. Other signatures of adaptation were also apparent. Genotypic variation in *A*_net_ and *g*_s_ was positively related to aridity under low precipitation conditions, and averaged across treatments, genotype intrinsic water‐use efficiency, and osmotic potential both declined with increasing aridity while genotype photosynthetic capacity (*V*_cmax_, *J*_max_) increased with aridity. The clinal patterns we observed demonstrate that genotypes from extremely arid environments exhibit a strategy of low water‐use efficiency, lower sensitivity to dry surface soils, and high photosynthetic capacity.

### 4.1 Clines in genotype responsiveness to water availability

An important result of our study was that home‐climate aridity predicted genotype responses to precipitation; genotypes from more arid locations were less responsive to reduced precipitation than genotypes from less arid locations. This was true of aboveground dry mass responses and to some extent, *Ψ*_pd,_ *A*_net,_ and *g*_s_ responses, although relationships between genotype responsiveness and aridity were not statistically significant for these traits. We expected to find greater drought tolerance or avoidance in more arid genotypes based on the hypothesis that genotypes sourced from the most arid locations would be adapted to dry surface conditions and would exhibit a phreatophytic habit with higher maximum rates of leaf gas exchange. Clinal patterns in responsiveness to water availability represents another attribute of local adaptation and genetic differentiation within species.

Our study is one of the few to identify clinal patterns in plant responsiveness to water availability. Pratt and Mooney (2013) also found clinal patterns in responsiveness to experimental precipitation treatments in *Artemisia californica*—an upland shrub species. In contrast to our results, *Artemisia californica* populations from the most arid sites were more responsive to increasing precipitation than populations from less arid sites. Similar patterns were also found in *Senna candolleana*, another upland shrub species (Lázaro‐Nogal et al., 2015). However, aridity–plasticity relationships appear more complex within other species (e.g., Carvajal et al., 2017; Welles & Funk, 2021), including broadly distributed trees (Aranda et al., 2014; Cooper et al., 2022; López et al., 2010; Meier & Leuschner, 2008). For example, McClean et al. (2014) found that leaf size and leaf thickness showed opposing patterns of plasticity in *Eucalyptus obliqua* populations sourced from an aridity gradient.

In nonphreatophytic tree species there is an expectation that arid genotypes will grow slower and possess more conservative stomatal behavior for avoiding low leaf water potentials under drying soils (López et al., 2016; Matías et al., 2014; Voltas et al., 2008). In *Eucalyptus camaldulensis*, a phreatophytic species, we find that arid genotypes tolerate rather than resist dry surface conditions. In this case, tolerance of surface drying was associated with acclimation of leaf hydraulic conductance (*K*_leaf_) and high rates of leaf gas exchange under dry conditions (Limousin et al., 2022). Other studies have also shown that *K*_leaf_ can acclimate to drying conditions in some genotypes and can help support leaf gas exchange under arid conditions (Martorell et al., 2015). It is important to note that arid genotypes are likely better able to avoid surface drought impacts by developing deep roots that access groundwater.

A limitation of our study is that we were unable to quantify root biomass or rooting depth of the genotypes. It is not uncommon for eucalypts growing in dry locations, including *E. camaldulensis*, to produce roots 5–10 m below the soil surface (Awe et al., 1976; Canadell et al., 1996; Christina et al., 2017; Dell et al., 1983). We hypothesize that genotypic variation in root production and rooting depth are major factors shaping our results, with genotypes from more arid environments investing in more roots that penetrate deeper into the soil profile (Garbowski et al., 2020). We find circumstantial evidence in support of this hypothesis. First, genotypes from more arid environments showed smaller reductions (lower response) in *Ψ*_pd_ under low precipitation than genotypes from less arid environments. This trend was weak but provided some evidence that arid genotypes had greater access to water under low precipitation. Second, across genotypes, we observed that *A*_net_ and *g*_s_ increased with aridity under low precipitation, and reductions in *A*_net_ and *g*_s_ under reduced precipitation were smaller in genotypes from more arid environments. In other arid zone phreatophytes, genotypes from low‐elevation hot environments transport water faster than genotypes from high‐elevation cool environments (Blasini et al., 2020). Under arid conditions, this may aid heat avoidance (Aparecido et al., 2020; Blasini et al., 2022). Third, we observed that more arid genotypes generally showed less aboveground growth and leaf area than less arid genotypes (Figure 1, Figure S4). Lower aboveground growth and lower leaf area in arid genotypes would reduce water demand and aid drought avoidance and could reflect greater allocation to roots. Indeed, a previous study in *E. camaldulensis* seedlings found that genotypes from more arid climates produced more fine roots and maintained higher rates of leaf gas exchange than genotypes from less arid climates (Gibson et al., 1995). Additional studies would be needed to explicitly compare rooting depth of these genotypes, and the degree to which rooting depth influenced drought tolerance and avoidance.

### 4.2 Aridity as a predictor of genotypic differences in leaf physiology

Averaged across treatments, several traits showed clinal relationships with aridity. Genotype means for Δ, *V*_cmax_, and *J*_max_ increased with aridity indicating lower intrinsic water‐use efficiency (*A*_net_/*g*_s_) but higher photosynthetic capacity in more arid genotypes than less arid genotypes. In some species, Δ declines with aridity or declining precipitation indicating higher intrinsic water‐use efficiency (Comstock & Ehleringer, 1992; Givnish et al., 2014). This pattern is interpreted as adaptive, reflecting tighter control of leaf water use in arid conditions. However, Δ can also be higher in species and genotypes from arid environments reflecting a less conservative water‐use strategy (Anderson et al., 1996; Pennington et al., 1999; Warren et al., 2006). Higher Δ in more arid genotypes fits with our interpretation of deeper roots, greater water access, higher leaf‐scale water use, and lower responsiveness to reduced precipitation in these genotypes. It is possible that high Δ in arid zone species and genotypes is reflective of semi‐obligate or obligate phreatophytic habit and heat avoidance (Hultine et al., 2020) where plants increase water loss regardless of carbon gain (Aparecido et al., 2020).

Our results could indicate that aridity has driven selection for higher photosynthetic capacity. Intraspecific variation in photosynthetic capacity has been observed in other studies (Aspinwall et al., 2011; Benomar et al., 2015), but to our knowledge, this is the first study to show a clinal pattern in photosynthetic capacity driven by aridity. Photosynthetic capacity, *N*_area_, and *R*_area_ generally increase along natural aridity gradients in Australia; however, these increases usually coincide with increased water‐use efficiency (Dong et al., 2017; Wright et al., 2001, 2005). We found that water‐use efficiency and photosynthetic capacity showed opposing relationships with aridity. These patterns suggest that lower water‐use efficiency in more arid genotypes was not due to lower photosynthetic capacity, but rather higher rates of leaf water loss (per unit C gain) integrated over time. Other studies within species have found positive genetic correlations between *V*_cmax_ and *g*_s_ (Bauerle et al., 2003; Geber & Dawson, 1997; Martin‐St. Paul et al., 2012), which could partly explain why water‐use efficiency decreased but photosynthetic capacity increased with aridity. Lower water‐use efficiency in more arid genotypes could also reflect increased conductance for CO_2_ diffusion to meet increased CO_2_ demand by Rubisco, resulting in increased water loss (Galmés et al., 2007; Medrano et al., 2002; Wong et al., 1979). Although water‐use efficiency and photosynthetic capacity increases across species growing along aridity gradients (e.g., Dong et al., 2017), our results suggest that these patterns do not entirely reflect adaptation and may not hold within species.

It is unclear why *V*_cmax_ and *J*_max_ increased with aridity across genotypes. High rates of *V*_cmax_ and *J*_max_ in arid zone species have been associated with high *N*_area_ and LMA (Hinojo‐Hinojo et al., 2018; Dong et al., 2020) and high respiratory costs (Atkin et al., 2015), yet we found no relationship between aridity and genotypic differences in *N*_area_, LMA, and *R*_area_. Variation in N allocation might explain why photosynthetic capacity and *N*_area_ were decoupled across genotypes (Funk et al., 2013; Takashima et al., 2004). Yet, previous studies across eucalypts have found no relationship between N allocation and aridity (Warren et al., 2006). Although average LS showed no relationship with aridity, further investigation revealed that *V*_cmax_ and average LS were positively correlated across genotypes (*r* ^2^ = .59, *p* = .02). This relationship could reflect a strategy for maximizing C uptake in arid environments when whole‐tree leaf area is low.

Our results also indicate that aridity is an important determinant of genetic differentiation in osmotic potential at full turgor (π_o_). This provides additional evidence of adaptation to aridity. More negative π_o_ help maintain cell turgor and leaf gas exchange under dry conditions (Bartlett et al., 2012). Similar patterns have been observed across related species (Fletcher et al., 2018), although we are unaware of other studies showing within species clines in π_o_. More negative values of π_o_ in more arid genotypes could partly explain why these genotypes showed smaller reductions in leaf gas exchange under low precipitation.

### 4.3 Genotype responses to water availability

We found that G × P was more common for hydraulic and gas‐exchange (process) traits than economic (pattern) traits. Averaged across genotypes, some economic traits (e.g., WD) did change with precipitation, but these changes were relatively small. Moreover, hydraulic and gas‐exchange trait responses to precipitation were better predictors of genotype productivity responses to precipitation. Hence, process traits that respond directly to changes in water availability are more responsive to soil moisture than economic traits, and genotypic variation in the responsiveness of these traits is predictive of genotype aboveground productivity responses to water availability.

Similar conclusions have been drawn when examining global‐scale relationships between functional traits and precipitation (Reich et al., 2007; Wright et al., 2004), community‐weighted functional trait responses to precipitation changes (Griffin‐Nolan et al., 2018), and trait responses to soil drying within individual species (López et al., 2010; Pritzkow et al., 2020; Westerband et al., 2019). Previous results from this experiment (Asao et al., 2020) also showed that pattern traits (LMA, leaf N, leaf P) were relatively insensitive to precipitation and were only weakly associated with genotype growth rate. Our study extends this work by showing that process traits are sensitive to water availability and the responsiveness of these traits differs among genotypes from more or less arid environments.

A caveat of our study is that low precipitation created a mild drying effect. Low precipitation reduced surface soil moisture and strongly reduced tree growth, but effects on average *Ψ*_pd_ and leaf gas exchange were relatively small (Table 2). The deep rooting nature of *E. camaldulensis* and occasional subsurface water movement into our plots (see Figure S2) could have influenced these results. Severe soil moisture reduction may create larger changes in pattern (e.g., economic traits), although we expect that process traits would still be more responsive to severe drying and better predictors of genotype productivity responses to precipitation.

As temperature and precipitation change around the globe, there is greater urgency to understand adaptation along aridity gradients, and genetic differentiation in traits and trait responses. Using a broadly distributed tree species, we show that genotypes sourced from an aridity gradient differ in growth, water use, and photosynthetic traits, as well as tolerance or avoidance of low water availability. The most arid genotypes possess a unique strategy defined by lower sensitivity to dry surface soils, low water‐use efficiency, and high photosynthetic capacity. This strategy is associated with lower aboveground growth and lower responsiveness to changing precipitation. Our results provide new insight into aridity as a driver of adaptation and shed light on the traits and trait responses that may improve plant performance in extremely arid environments.

## AUTHOR CONTRIBUTIONS

Michael J. Aspinwall, Paul D. Rymer, Mark G. Tjoelker, and David T. Tissue designed the experiment. Michael J. Aspinwall lead the data collection, analysis, and writing with help from Jeff Chieppa. Chris J. Blackman, Danielle Creek, and Robert J. Griffin‐Nolan collected leaf water potential and leaf hydraulics data. Chelsea Maier managed the experimental infrastructure and collected growth and micrometeorological data. All authors contributed to the writing and data interpretation.

## Supporting information


Data S1

## Data Availability

The data that support the findings of this study are openly available in Figshare: https://doi.org/10.6084/m9.figshare.19747135.v1.
