# Supplementary material for: Aridity drives clinal patterns in leaf traits and responsiveness to precipitation in a broadly distributed Australian tree species
Source: Plant Environ Interact. 2023 Mar 17;4(2):70–85. doi: 10.1002/pei3.10102 (PMC10243541; doi:10.1002/pei3.10102)
Supplement: Supplementary file 1 — Data S1 [file PEI3-4-70-s001.zip › PEI3_10102_Aspinwall_SIEF_ROS_Supplemental.docx]

**Supporting Information**

**Title: Aridity drives clinal patterns in leaf traits and responsiveness to precipitation in a broadly distributed Australian tree species**

Michael J. Aspinwall^1,2,3*^, Chris J. Blackman^1,4^, Chelsea Maier^1^, Mark G. Tjoelker^1^, Paul D. Rymer^1^, Danielle Creek^1,5^, Jeff Chieppa^2^, Robert J. Griffin-Nolan^6^, and David T. Tissue^1,7^

*^1^Hawkesbury Institute for the Environment, Western Sydney University, Locked Bag 1797, Penrith NSW 2751, Australia*

*^2^College of Forestry and Wildlife Sciences, Auburn University, Auburn, AL 36849 USA*

*^3^Formation Environmental, LLC, Sacramento, CA 95816*

*^4^ARC Centre of Excellence for Plant Success in Nature and Agriculture, School of Natural Sciences, University of Tasmania, Hobart 7001, Australia*

*^5^Faculty of Environmental Sciences and Natural Resource Management, Norwegian University of Life Sciences (NMBU), Ås, Norway*

*^6^Department of Biological Sciences, California State University, Chico, CA 95929 USA*

*^7^Global Centre for Land Based Innovation, Western Sydney University, Hawkesbury Campus, Richmond NSW 2753, Australia*

*Corresponding author, email: mjaspinwall@gmail.com, phone: +1-(904)-891-2794

**Methods S1.**

***Growth, biomass, and economic traits***

Trees were harvested the week of 6 March 2017, roughly 650 days (1.75 yrs) after the treatments began. Average stem length at harvest was > 5 m. At harvest, diameter at breast height (1.3 m, DBH) was recorded and the shoot of each tree was cut at ground level. Total fresh mass of each component was immediately measured using a balance. We estimated total dry mass of each component by collecting, weighing, and drying subsamples to determine dry matter content. For leaves, a random subsample of 100 leaves per tree were collected and fresh mass was recorded. Surface area (m^2^) of these leaves was determined using a leaf area meter (LI-3100C, LiCor Inc., Lincoln, NE, USA). Average leaf size (LS) per tree was calculated as subsample surface area divided by 100. Leaves were dried at 70 °C for three days and leaf dry mass per unit area (LMA, g m^-2^) was estimated by dividing leaf subsample dry mass by leaf area. Total leaf dry mass (leaf DM) was estimated by multiplying the subsample dry matter content by total leaf fresh mass. Total leaf area (LA, m^2^) was estimated by multiplying subsample leaf area per unit dry mass (m^2^ g^-1^) by the estimate of leaf DM.

Branch dry mass (branch DM) was determined by collecting, weighing, and drying (70 °C, 7 days) a subsample of branches (~25% of total branch fresh mass), and multiplying subsample dry matter content by branch fresh mass. Stem dry mass (stem DM) was determined by collecting, weighing, and drying (70 °C, 7 days) a subsample of stem material (3-5 cm thick sections collected at 30-50 cm intervals), and multiplying subsample dry matter content by stem fresh mass. Wood density of each tree was determined at 25 cm of stem length. We cut and removed the bark from a thin (~1 cm) disk and averaged four measurements of disk diameter and thickness to estimate disk volume (cm^3^) based on the volume of a cylinder. The disk was dried at 70 °C for seven days and dry mass was divided by volume to estimate wood density (WD, g cm^-3^). No heartwood had formed in these young trees, so measurements of disk diameter were used to estimate sapwood area (SA, m^2^) based on the area of a circle. Huber value (HV × 10^4^) of each tree was calculated as the ratio of SA to total tree leaf area.

A subsample of dried leaf material from each tree was ground and analyzed for leaf C and N content (%) using a combustion elemental analyzer (CE Instruments, Wigan, UK). Nitrogen per unit leaf area (*N*_area_, g N m^-2^) was calculated as the product of %N and LMA. The isotopic composition of leaf C (δ^13^C, ‰) was measured using an Isochrom continuous-flow mass spectrometer (Micromass, Manchester, UK). Discrimination of ^13^C relative to the atmosphere (Δ) was calculated following (Farquhar *et al.,* 1989), assuming the δ^13^C of atmospheric CO_2_ was -8‰. Δ is inversely related to intrinsic water use efficiency.

**Methods S2.**

***Leaf hydraulic traits***

Leaf hydraulic vulnerability was assessed in February 2017 using a single-point approach (Lucani *et al.,* 2018), where leaf hydraulic conductance (*K*_leaf_, mmol m^-2^ MPa^-1^ s^-1^) was measured at a reference water potential associated with embolism-induced hydraulic decline. This allowed us to compare *K*_leaf_ across genotypes and treatments without determining the response of *K*_leaf_ to the full range of water potentials. We targeted a reference water potential of -4 MPa to make comparisons of dehydrated *K*_leaf_ and relative hydraulic vulnerability. This level of water stress was associated with incipient *K*_leaf_ decline in a series of unpublished leaf hydraulic vulnerability curves for *E. camaldulensis*. This level of water stress was associated with incipient *K*_leaf_ decline in a series of unpublished leaf hydraulic vulnerability curves for *E. camaldulensis*.

*K*_leaf_ was measured using the timed rehydration kinetics technique (Brodribb & Holbrook 2003; Blackman & Brodribb 2011). Two branches with attached leaves ~60 cm in length were sampled from a single tree of each genotype and treatment within each of three shelters (*n*=108 branches). Branches were sampled in late afternoon and transported to the lab where they were recut underwater with the cut end submerged overnight to reach full hydration. In the morning, branches were removed from water and slowly dehydrated during which time leaves were regularly sampled for leaf water potential determination. At a target water potential of -1.5 MPa, which precedes *K*_leaf_ decline in the reference vulnerability curves, one branch per tree was placed inside a humidified plastic bag to arrest further water potential decline. These branches were used to calculate maximum *K*_leaf_ (see below). The remaining branch per tree was dehydrated over the next 24 hrs until reaching -4 MPa. These branches were used to calculate dehydrated *K*_leaf_.

The water potential of branches at each target water potential was remeasured using a neighboring leaf, after which a sample leaf was excised under water in a shallow dish and allowed to rehydrate through the petiole. After a rehydration period of 20 seconds for branches at *ca*. -1.5 MPa and 90 seconds for branches at *ca*. -4 MPa, the leaf was removed from water and the cut end quickly dabbed dry. The final water potential of the sample leaf was determined and *K*_leaf_ calculated from the ratio of the initial and final water potential and the capacitance of the leaf:

*K*_leaf_ = *C*_leaf_ ln[*Ψ*_o_/*Ψ*_f_]/*t*

where *Ψ*_o_ is the initial leaf water potential (MPa); *Ψ*_f_ is the final water potential (MPa); *t* is the duration of rehydration (s); and *C*_leaf_ is leaf capacitance (mmol m^-2^ MPa^-1^). Leaf capacitance was calculated from the pre- and post-turgor loss slope of the pressure-volume curve (see below) multiplied by the saturated mass of water in the leaf and LMA. For each sample leaf, *K*_leaf_ was calculated using genotype and treatment level *C*_leaf_ means.

**A**
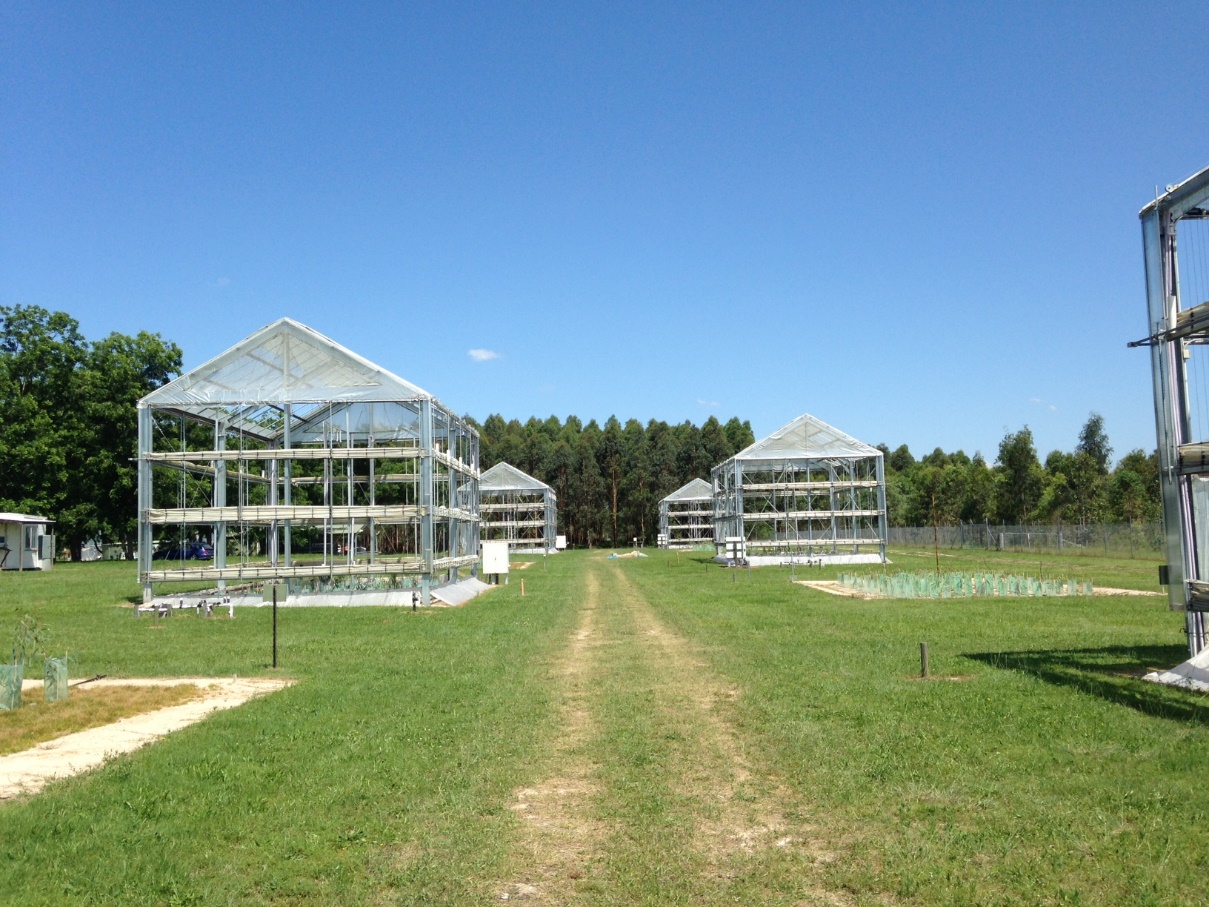


**B**
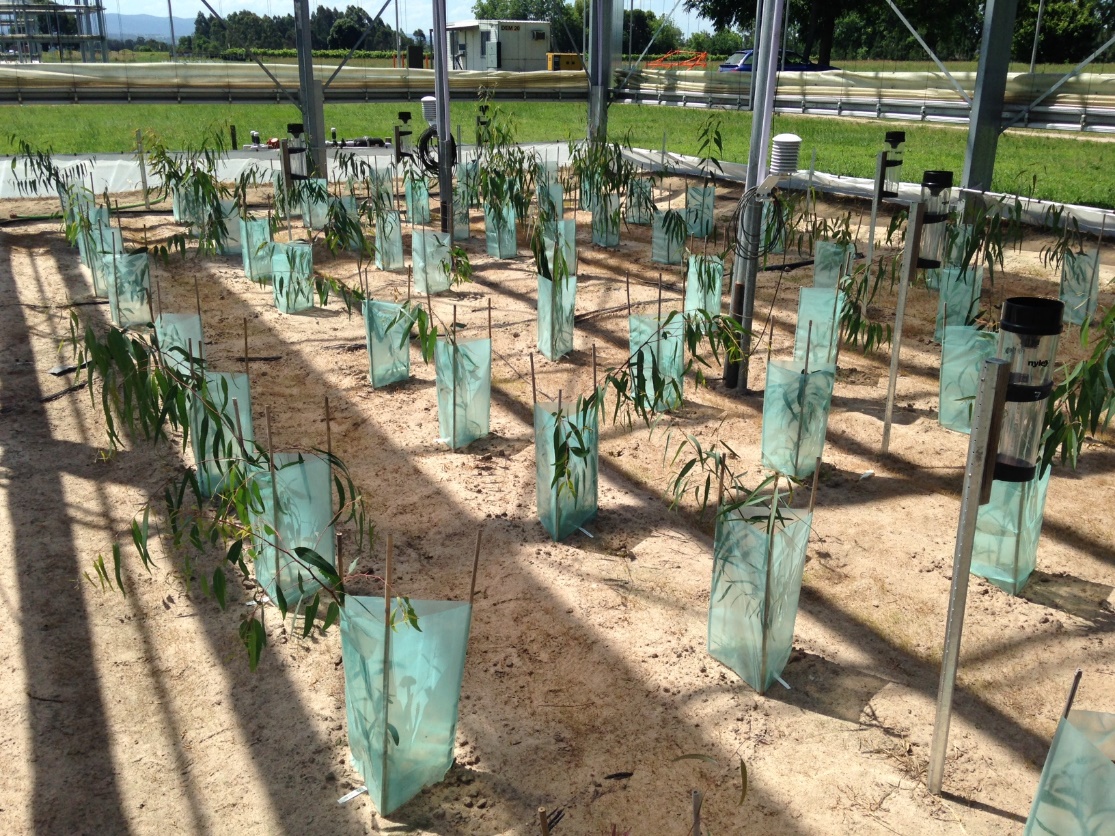


**C
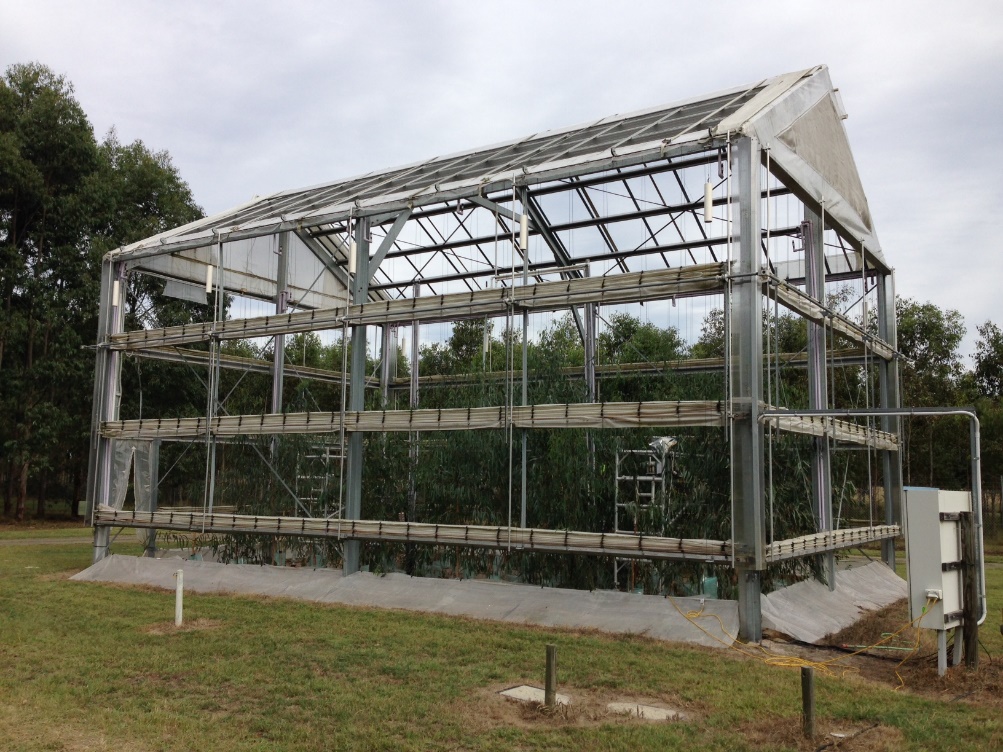
**

**Fig S1.** (a) Photo of rainout shelters (ROS) on campus of Western Sydney University in Richmond, NSW. (b) Photo showing internal layout of trees just after transplanting into the ROS. (c) Close-up photo of ROS infrastructure at the end of the experiment with full-size trees.


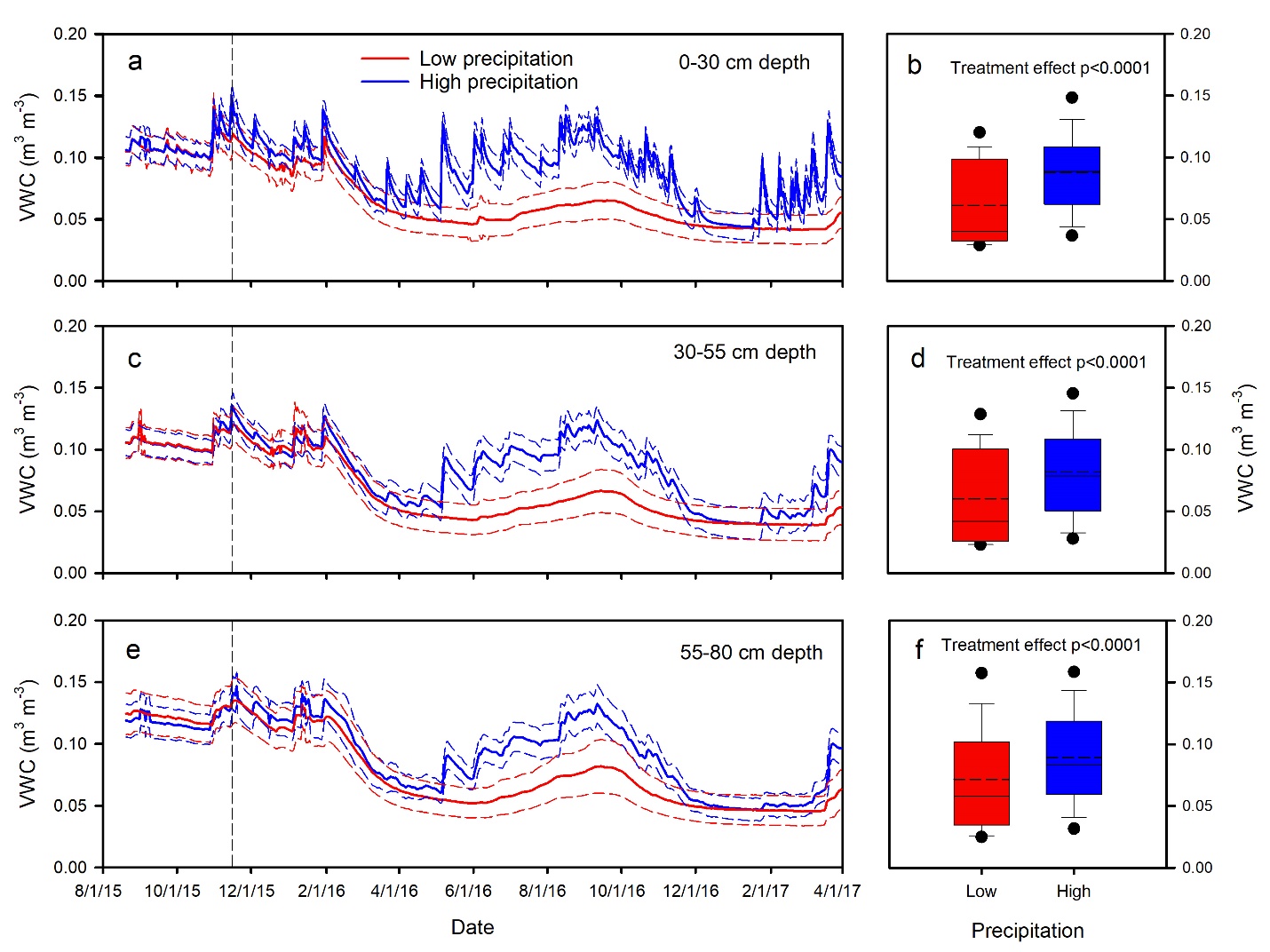


**Fig S2.** (a,c,e) Mean daily soil volumetric water content (VWC) over time in low and high precipitation treatments at a rainout shelter facility in Richmond, NSW, Australia. Solid lines are mean values and dashed lines are 95% confidence intervals. The dashed vertical line occurring on 15 November 2015 marks the start of the treatments (b,d,f) Box-plots for mean daily VWC in high and low precipitation treatments during the experimental period (15 November 2015 – April 2017). The boxes represent the interquartile range (25th–75th percentile) of VWC in each treatment. The lower and upper whiskers represent the 10th and 90th percentiles, respectively. Filled circles represent VWC values outside the 10th and 90th percentiles. Within each box plot, the overall mean VWC is shown as a dotted line, and median VWC is shown as a solid line.


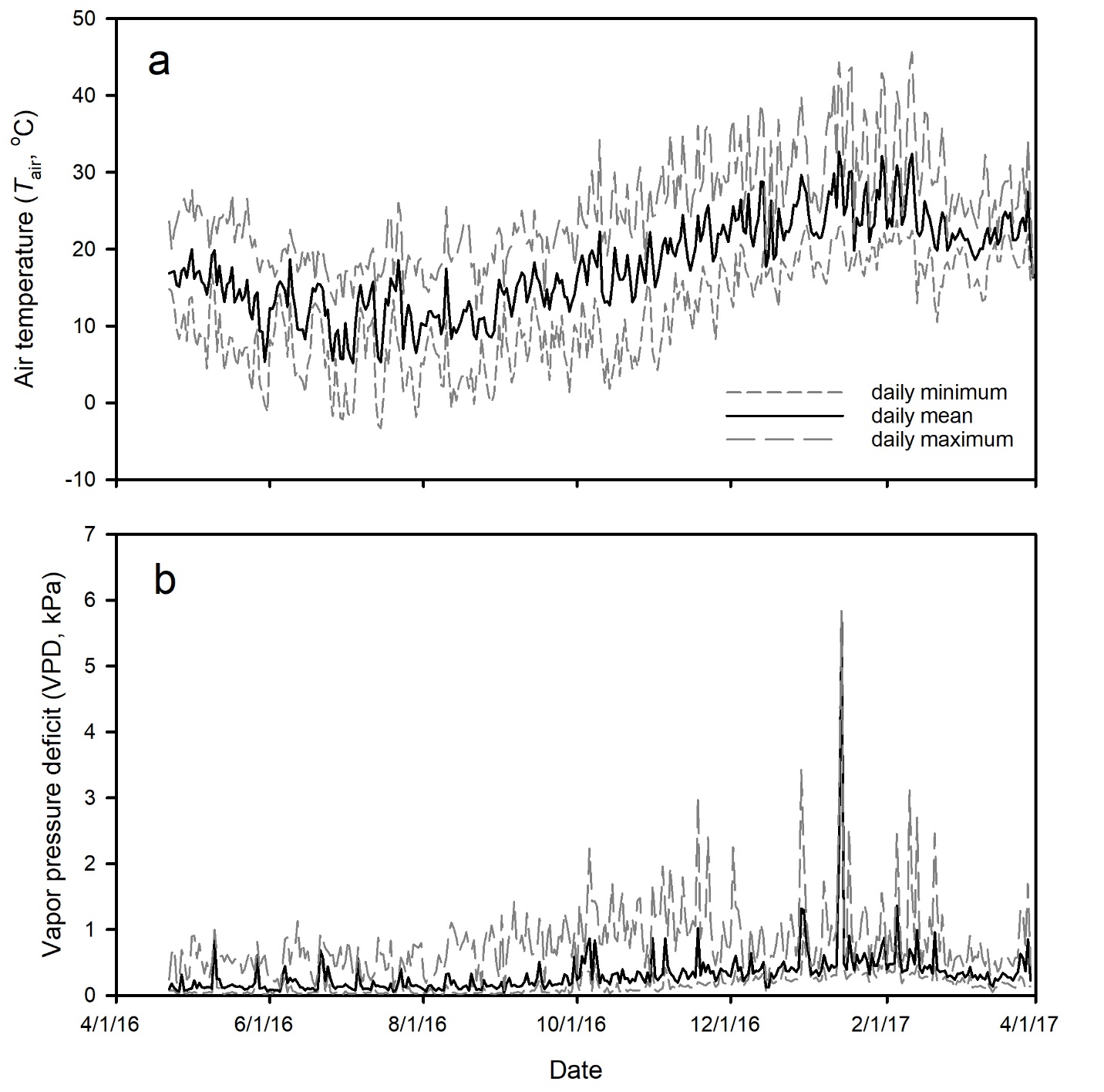


**Fig S3.** (a) Daily mean minimum, mean, and mean maximum air temperature over time averaged across six rainout shelters in Richmond, NSW, Australia. (b) Daily mean minimum, mean, and mean maximum vapor pressure deficit over time.


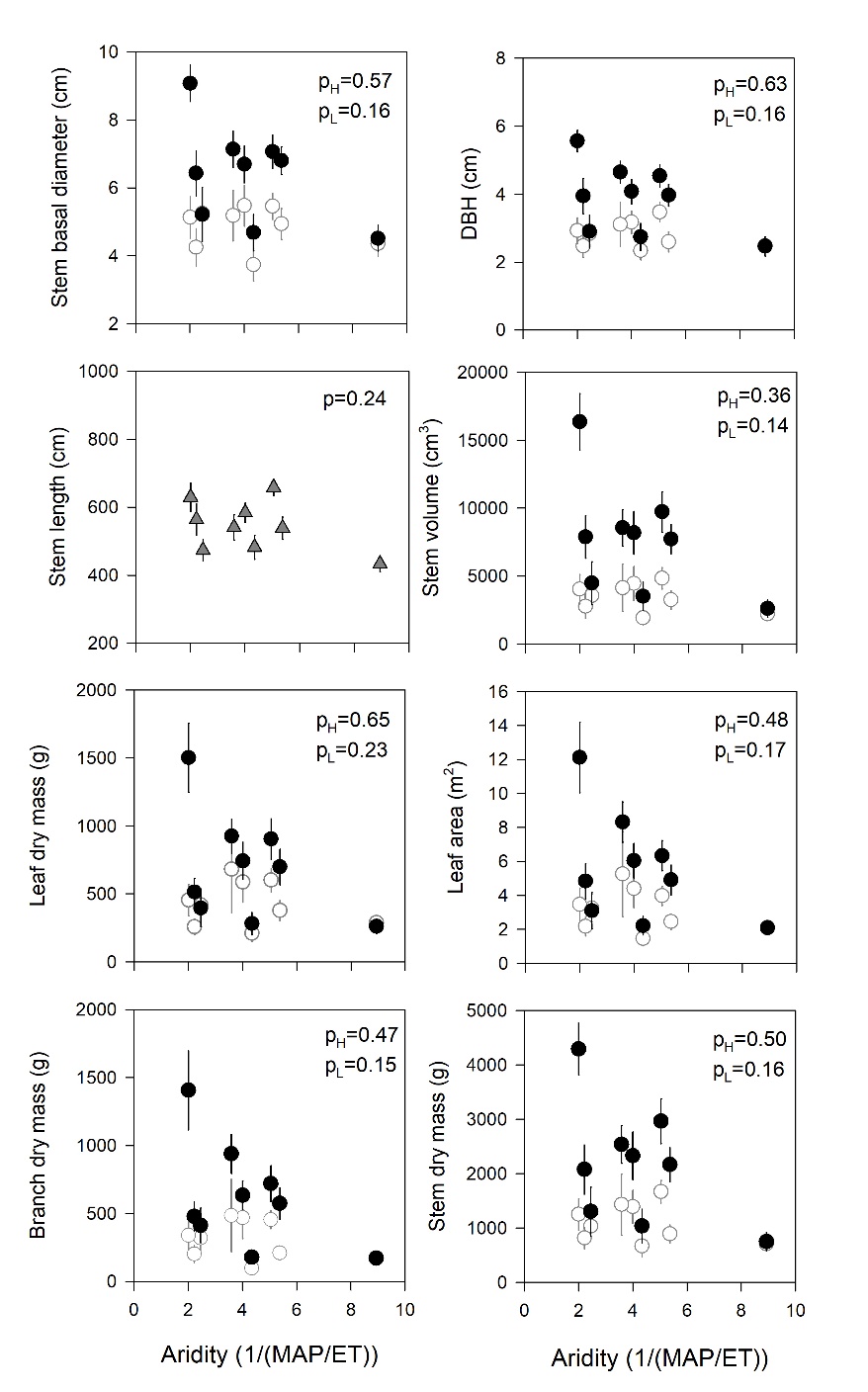


**Fig S4.** The relationship between *Eucalyptus camaldulensis* subsp. *camaldulensis* home-climate aridity and several variables describing aboveground growth or dry mass production. Filled circles are genotypes means (±standard error, n=6) for growth/dry mass under high precipitation. Open circles are genotype means (±standard error, n=6) for growth/dry mass under low precipitation. Grey triangles are genotype means (±standard error, n=12) for growth/dry mass averaged across low and high precipitation treatments. Probability values are shown in each panel and indicate the significance of the relationship between aridity and genotype means.

**
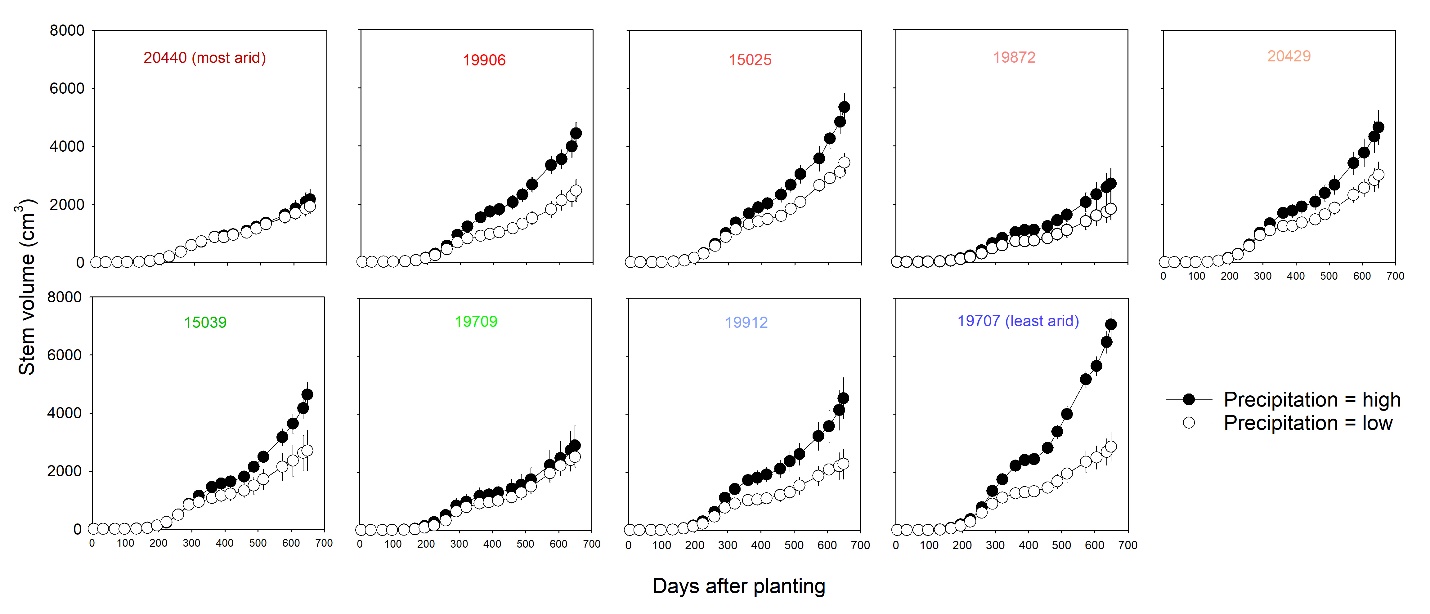
**

**Fig S5** Mean stem volume (±standard error, *n*=6) of different *Eucalyptus camaldulensis* subsp. *camaldulensis* genotypes (codes indicate seedlot numbers) over time under high and low precipitation treatments in a rainout shelter facility in Richmond, NSW, Australia. Genotypes are arranged from most arid (20440) to least arid (19707) origin.

**
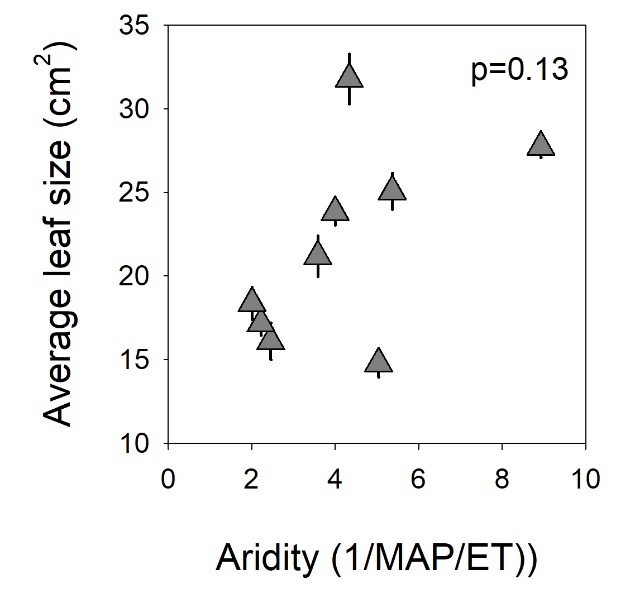
**

**Fig S6.** Relationship between home-climate aridity and genotype average leaf size (± standard error, *n*=12) in *Eucalyptus camaldulensis* subsp. *camaldulensis*. Data are averaged across precipitation treatments.
